# The Effect of Controlled Diabetes and Hyperglycemia on Implant Placement with Simultaneous Horizontal Guided Bone Regeneration: A Clinical Retrospective Analysis

**DOI:** 10.1155/2021/9931505

**Published:** 2021-06-14

**Authors:** Paolo De Angelis, Paolo Francesco Manicone, Giulio Gasparini, Ilaria De Filippis, Margherita Giorgia Liguori, Silvio De Angelis, Francesca Cannata, Antonio D'Addona

**Affiliations:** ^1^Department of Head and Neck, Division of Oral Surgery and Implantology, Institute of Clinical Dentistry, Fondazione Policlinico Universitario A. Gemelli IRCCS—Università Cattolica del Sacro Cuore, Rome, Italy; ^2^Department of Head and Neck, Division of Oral and Maxillofacial Surgery, Institute of Clinical Dentistry, Fondazione Policlinico Universitario A. Gemelli IRCCS—Università Cattolica del Sacro Cuore, Rome, Italy; ^3^Ascoli Piceno, Italy

## Abstract

Diabetes represents a challenge in implant therapy because hyperglycemia may negatively affect bone regeneration, directly compromising clinical outcomes and increasing clinical failures. The aim of this retrospective study is to analyse the prognostic significance of HbA1c levels in patients undergoing implant placement associated with horizontal guided bone regeneration. Thirty-four patients were divided into 3 groups according to their HbA1c levels: nondiabetic normoglycemic patients (HbA1c < 5.7%), nondiabetic hyperglycemic patients (HbA1c < 6.5%), and controlled diabetic patients (HbA1c < 7%). Primary outcomes were dimensional changes in height (VDH) and width (DW) of the peri-implant defect. Secondary outcomes were evaluations of periodontal parameters of adjacent tooth sites, wound healing, marginal bone loss (MBL), and survival and success rates. At *T*_1_ (6 months), mean VDH values in groups 1, 2, and 3 were, respectively, 0.07, 0.5, and 0.25 mm. Mean DW values in those same groups were, respectively, 0.07, 0.38, and 0.33 mm. HbA1c levels were not statistically related to VDH and DW values at *T*_1_. No statistically significant differences were observed in MBL between groups (*p* = 0.230). Implant survival and success rates were, respectively, 98% and 96%. Simultaneous guided bone regeneration is a feasible procedure for the treatment of horizontal bone deficiencies in controlled diabetic patients.

## 1. Introduction

Diabetes is a group of metabolic diseases characterized by hyperglycemia resulting from defects in insulin secretion, insulin action, or both [1]. It is one of the most critical public health problems and the main cause of morbidity and mortality in modern societies. Recent data reveal that diabetes mellitus is increasing at an alarming rate in many countries, and it is estimated that 450 million people have the disease. Moreover, with the current striking plateauing of diabetes mellitus in adults, it is estimated that the rates may increase up to 71.1 million by 2040 in Europe [2]. The vast majority of diabetes cases fall into two main etiopathogenetic categories: type 1and type 2 [1].

The risk of developing type 2 diabetes increases with age, obesity, and lack of physical activity. It is often associated with a strong genetic predisposition or family history in first-degree relatives, more than type 1 diabetes. However, the genetics of type 2 diabetes are poorly understood [3].

In both type 1 and type 2 diabetes, various genetic and environmental factors can result in the progressive loss of *β* cell mass and/or function that manifests clinically as hyperglycemia. Once hyperglycemia occurs, patients with all forms of diabetes are at risk for developing the same chronic complications, although the rates of progression may differ [3]. Enhanced blood glucose levels in chronic hyperglycemia increase the formation and accumulation of advanced glycation end products (AGEs), and the interaction between AGEs and their receptor, RAGE, plays a key role in the development of complications [4].

Diabetes mellitus has also been associated with the occurrence of a series of complications involving the skeletal system, collectively referred to as *diabetic bone disease* or *diabetic osteopathy* [5]. The diabetic skeletal phenotype presents the following features:

- Decreased linear bone growth during the pubertal growth spurt in adolescents with diabetes [6, 7]
- Reduced bone mineral density and increased risk for osteopenia and osteoporosis [8]
- Increased fracture risk [9]
- Poor osseous healing characteristics and impaired bone regeneration potential [10–12]

Clinical and in vivo studies have established that impaired intramembranous and endochondral ossifications constitute dominant pathophysiological traits characterizing diabetic bone disease [13].

The currently available evidence seems to support the premise that hyperglycemia and/or hyperinsulinemia are the main mechanisms underlying diabetic bone pathophysiology [14].

Guided bone regeneration (GBR) was introduced as a therapeutic modality aiming to achieve bone regeneration by the use of barrier membranes [15–18]. From the evidence obtained through a literature search, no clinical study has assessed guided bone regeneration outcomes in people with a wide range of glycemic control, and studies are required in all major ethnic groups to establish more precisely the glycated haemoglobin (HbA1c) levels predictive of complications.

It should be considered that 30% of patients aged ≥30 years seen in general dental practices have dysglycemia. The rising number of diabetic patients and patients with dysglycemia represents a challenge for the high number of procedures involving bone replacement or augmentation, as hyperglycemia may delay and/or impair bone regeneration, directly compromising clinical outcomes and increasing clinical failures [13, 19].

The present study is aimed at assessing the prognostic significance of the glycated haemoglobin (HbA1c) levels in patients undergoing implant placement associated with horizontal GBR, as well as the correlation between glycemic control levels and clinical findings.

## 2. Materials and Methods

The study population consisted of all patients requiring implant placement associated with horizontal guided bone regeneration, who had been treated in the Oral Surgery and Implantology Department of the Catholic University in Rome. All reported investigations were carried out in accordance with the 1975 Helsinki Declaration, as revised in 2013 for ethical approval. All participants provided a written informed consent after being thoroughly informed about the study's objectives and procedures. Because of the retrospective nature of this study, it was granted an exemption in writing by the local ethics committee. All surgeries were performed by the same trained and experienced surgeon, and all clinical measures were recorded by the same examiner.

Inclusion criteria were as follows:

- Patients in need of one (or more) implants in the upper or lower jaw
- Patients in need of horizontal bone augmentation
- Bone defects which allowed us to obtain an adequate primary stability with a bone dehiscence that could be treated using a resorbable membrane
- FMPS and FMBS below 15%
- Age > 20 years
- HbA1C levels recorded in the clinical chart

Patients were excluded in the presence of any of the following conditions:

- General contraindications for implant placement and/or surgical treatment
- Uncontrolled periodontal disease
- Any drug or medication known to affect oral status and bone turnover or contraindicate surgical treatment (e.g., immunosuppressant, corticosteroid, or bisphosphonate therapy)
- History of malignancy, radiotherapy, or chemotherapy for malignancy
- Smoker
- Blood-related diseases
- Excessive alcohol consumption
- Conditions associated with an altered relationship between HbA1C and glycemia such as sickle cell disease, pregnancy, glucose-6-phosphate dehydrogenase deficiency, HIV, haemodialysis, recent blood loss or transfusion, or erythropoietin therapy
- Unwillingness to return for follow-up examinations

### 2.1. Detected Parameters

The following data were collected from the clinical chart:

- Age
- Sex
- Race
- Height and weight
- Oral hygiene maintenance therapy (yes: at least one prophylaxis per year; no: less than one per year or none)
- Osteopenia/osteoporosis (yes/no)

Based on the case history of diabetes and the levels of HbA1c on admission, patients were divided into three groups:

- Nondiabetic normoglycemic patients (HbA1c < 5.7%)
- Nondiabetic hyperglycemic patients (HbA1c < 6.5%)
- Controlled diabetic patients (HbA1c < 7%)

The body mass index (BMI) of individuals in all groups was calculated by estimating the weight in kilograms (kg) and height in square metres (m^2^), which were recorded in the patients' charts. The pharmacological therapy of each patient was recorded as well.

The peri-implant osseous defect was measured after implant placement using a periodontal probe and the following parameters were recorded in the clinical chart as previously described by Jung et al. [20]:

- Vertical defect height (mm) measured from the implant shoulder to the first bone-to-implant contact (BIC)
- Infrabony defect height (mm) measured from the bone crest to the first BIC
- Defect width (mm) measured from the mesial to the distal bone crests at the level of the implant shoulder
- Horizontal defect depth (mm) measured from the bone crest to the implant surface in a direction perpendicular to the long axis of the implant

Clinical evaluations have been performed at baseline (*T*_0_), 1 and 3 weeks, and 3, 6 (*T*_1_), and 12 months (*T*_2_) after surgery. Every patient underwent preoperative cone-beam CT scan with a resolution of 100 *μ*m in order to complete the preoperative planning and evaluate the bone height and thickness of the cortical plates.

A cone-beam CT scan was taken at six months of healing. Primary outcomes were the measurement of dimensional changes in ridge width (mm) and height (mm) at six months after the procedure, assessed using a periodontal probe during the second-stage surgery. Secondary outcomes were the evaluations of periodontal parameters of the tooth sites adjacent to the treatment areas. The widths of keratinized tissue (KT), plaque index (PI), gingival index (GI), probing depth (PD), and bleeding on probing (BOP) were also measured at the tooth sites adjacent to the treatment areas at *T*_0_ and *T*_1_ and recorded.

The occurrence of adverse events (e.g., wound infection, exposure of the graft and soft tissue dehiscence, and necrosis) was recorded during the whole duration of the follow-up. Wound healing was assessed using the early wound healing score (EHS), which is composed of 3 parameters: clinical signs of reepithelialization, clinical signs of haemostasis, and clinical signs of inflammation. The summation of the points of these 3 parameters generates the EHS. The EHS for ideal wound healing is 10 points, while the worst possible score is 0 points. Recordings of EHS were performed every seven days for the first three weeks [21].

Marginal bone loss (MBL) was assessed immediately after prosthesis delivery and at 12 months from prosthesis delivery with intraoral radiography, utilizing the long cone parallel technique. A bite made of silicone (3M™ Express, 3M ESPE Dental Products, St. Paul, MN, USA) was placed in the holding system, allowing for it to be repositioned precisely during each follow-up visit. Linear measurements (mm) on the digital images were performed to record the distances of the most coronal points in the mesial and distal ridge aspects from the implant shoulder.

### 2.2. Surgical Procedures

Before surgery, patients received antibiotic therapy (2 × 1 g amoxicillin clavulanate). The perioral skin was disinfected by means of a sterile gauze mounted on Klemmer forceps and soaked in povidone-iodine solution. Patients were then covered with TNT drapes, leaving only the oral cavity uncovered; mucous membranes were cleaned with a gauze soaked in 0.2% chlorhexidine.

Surgery was performed under local anaesthesia (articaine 4% with epinephrine 1 : 100,000). The horizontal incision was placed crestal in the lower jaw and slightly buccal on the upper jaw, extending from the distal aspect of the mesial tooth to the mesial aspect of the distal tooth. The incision was continued intrasulcularly in both the buccal and lingual areas. Releasing incisions were performed at the buccal, mesial, and distal line angles. A mucoperiosteal flap was raised, and the bone was exposed and carefully curetted. The adjacent teeth were carefully cleaned using ultrasonic and manual instruments. The insertion of the bone level implants, with lengths between 8 and 12 mm and diameters between 4.1 and 4.8 mm, was carried out according to the manufacturer's protocol. During implant placement, primary stability was assessed via insertion torque and hand testing. Measurements of the defect were performed using a periodontal probe (UNC-15). The cortical plate was perforated by means of a round bur to favour bleeding and access to the marrow cavity. Periosteal releasing incisions were used to allow tension-free adaptation of the mucoperiosteal flaps. A resorbable collagen membrane was shaped according to the recipient site and fixed on the lingual/palatal side with two or three fixation pins. The autogenous bone chips were collected from the areas surrounding the peri-implant defect using a bone scraper; they were then placed adjacent to the implant surface and mixed with deproteinised bovine bone mineral using a 50 : 50 ratio to fill the defect area completely. The membrane was closed over the graft and fixed on the buccal side using two or three titanium pins. The crestal incision was sutured with PTFE internal horizontal mattress sutures; finally, PTFE single sutures were placed on the vertical incisions and between the mattress sutures. Patients were instructed to rinse twice a day with 0.2% chlorhexidine mouth rinse and to continue the antibiotic regimen for 6 days. In addition, analgesics (500 mg ketoprofen) were prescribed for the next 3 days, according to individual needs. Patients were also instructed to refrain from mechanical plaque removal in the area for 2 weeks and to rinse twice daily with a 0.2% chlorhexidine mouth rinse. Sutures were removed 21 days following surgery. All patients were enrolled in a maintenance care program. The second surgery was carried out after six months.

### 2.3. Statistical Analysis

Descriptive statistics used for continuous factors included means and SDs, and medians and interquartile ranges (IQRs); in the case of categorical factors, absolute and relative frequencies (%) were employed. In order to assess whether glycemic status in patients with horizontal bone defects represents a factor capable of influencing clinical outcomes, patients were classified as “successes” and “non-successes,” where success meant the achievement at *T*_1_ of a vertical defect height and width equal to 0 mm. Correlations between categorical variables were made by using the chi square or Fisher exact test, while those between continuous variables were calculated by means of the Mann–Whitney *U* test.

Binary logistic regression was adopted to test the effects of the considered variables, treating the indication success/non-success as a dependent variable. In order to evaluate the possible influence of glycemic level on clinical results, we proceeded with a two-way mixed ANOVA model. A two-tailed value of *p* < 0.05 was considered significant. All analyses were conducted using the Stata version 14.2 software program (StataCorp, College Station, TX, USA).

## 3. Results

The study sample consisted of 34 patients (15 women and 19 men; mean age: 69.56 years; SD: 8.2 years). Fourteen patients (mean age: 71 ± 7 years; 7 females and 7 males) were included in group 1, 8 patients (mean age: 72 ± 5 years; 3 females and 5 males) were included in group 2, and 12 patients (mean age: 66 ± 9 years; 5 females and 7 males) were included in group 3. All participants received implant placement and simultaneous horizontal GBR between 1 January 2017 and 1 January 2019. Patients in group 3 were all affected by type 2 diabetes. In total, 50 implants were placed. Twenty-one participants (62%) received a single implant, while 13 (38%) received multiple implants (Table 1). All of the surgeries were successfully carried out, no intraoperative complications were recorded, and all implants obtained an adequate primary stability (insertion torque ≥ 25 Ncm).

### 3.1. Complications

Only one patient belonging to group 2 experienced a case of early implant loss, which was successfully replaced after three months, and two patients presented biological complications during the first 3 weeks after surgery.

All of the complications were cases of wound dehiscence, which were treated by local disinfection (rinsing with 0.2% chlorhexidine mouth rinse and applying 1% chlorhexidine gel), and all affected patients recovered completely after 2–3 weeks. Overall implant survival and success rates were, respectively, 98% and 96%. The implant survival rates were 100% in group 1, 93% in group 2, and 100% in group 3.

### 3.2. Clinical and Radiographical Parameters

28 patients (82.4%) were classified as “success” and 6 patients (17.6%) as “no-success,” where success meant the achievement at *T*_1_ of a vertical defect height and width equal to 0 mm. The number of implants was placed, and the infrabony defect height and the EHS were the only variables statistically significant (*p* < 0.05).

Binary logistic regression outlined that the variable horizontal defect depth (HDD) was the only one statistically significant to obtain a successful result, which was considered to be the complete defect closure (*p* = 0.009, OR = 37.6 [95%CI = 2.5–563.2]), indicating that an increase of the HDD is associated with a higher success rate.

**Figure 1:**
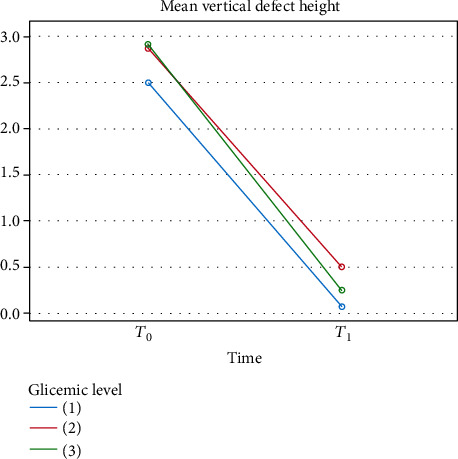
Vertical defect height changes from *T*_0_ to *T*_1_ for each group.

**Figure 2:**
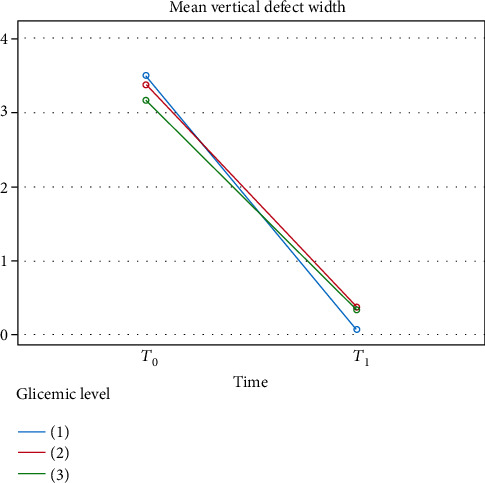
Defect width changes from *T*_0_ to *T*_1_ for each group.

After six months, statistically significant reductions in vertical defect height (VDH) and defect width (DW) were recorded in all groups in comparison to the time of baseline evaluation (*p* < 0.05) (Figures 1 and 2). In detail, at *T*_0_, the mean defects' heights in groups 1, 2, and 3 were, respectively, 2.50, 2.88, and 2.92 mm. In group 1, only one patient had a residual vertical defect height, while in group 2, there were two cases, and in group 3, there were three cases. The mean residual defect heights in groups 1, 2, and 3 were, respectively, 0.07, 0.5, and 0.25 mm, with no statistically significant difference between the groups (*p* = 0.187) (Table 2).

Mean defect widths were 3.50 mm in group 1, 3.38 mm in group 2, and 3.17 mm in group 3 at the baseline, while they were 0.07, 0.38, and 0.33 mm at *T*_1_. Mean residual defect widths of the groups also showed no statistically significant difference between the groups (*p* = 0.902) (Table 3).

KT values at *T*_0_ were significantly different from KT values at *T*_1_. No statistically significant differences in postoperative width of keratinized mucosa were observed between the groups (*p* = 0.499).

**Figure 3:**
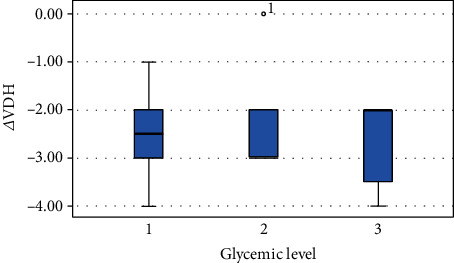
Difference of vertical defect height from *T*_0_ to *T*_1_ for each group.

**Figure 4:**
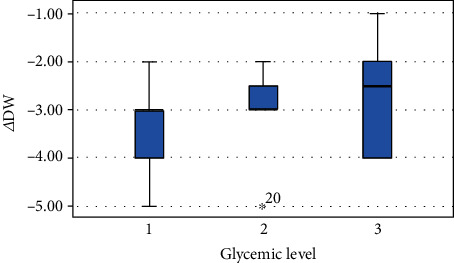
Difference of defect width from *T*_0_ to *T*_1_ for each group.

HbA1c levels were not statistically related to ΔVDH and ΔDW (*p* = 0.519; *p* = 132). These variables showed a weak correlation with glycemic levels. In particular, for the variables ΔVDH and ΔDW, the best clinical outcomes occurred at low HbA1c levels (Figures 3 and 4).

No statistically significant differences were observed in peri-implant marginal bone loss (MBL) between the groups (*p* = 0.230), and none of the patients displayed marginal bone resorption of more than 1.5 mm at 12 months from prosthesis delivery.

Probing depth, bleeding on probing, and width of keratinized mucosa showed no significant differences at *T*_1_ between the three groups (*p* = 0.418).

No statistically significant differences in postoperative wound healing were observed for the first 3 weeks between the groups analyzing the EHS (*p* > 0.05).

## 4. Discussion

The worldwide incidence of diabetes is increasing at a rapid rate [22]. This trend should be considered of clinical relevance by clinicians, especially in an ageing population, in relation to the placement and maintenance of oral implants. The results of various studies suggest that dental implants may be placed in diabetic patients with favourable outcomes, if glycemic status is within control ranges [23, 24] and patients are enrolled, after an accurate selection, in strict pre-, intra-, and postoperative programs [25, 26].

However, this disease represents a challenge because hyperglycemia may negatively affect bone regeneration, directly compromising clinical outcomes and increasing clinical failures [27]. This topic is relevant also because during an ordinary preparation of the osteotomic site or implant insertion, unplanned bone dehiscences or fenestrations may frequently occur in the diabetic and require an augmentation procedure in order to not leave the implant surface exposed [28].

Diabetic patients may undergo complications related to the surgical procedure and the early postoperative phase, as well as to the long-term maintenance of the implants [29]. The first complications are related to surgery and mainly consist in impaired wound healing and decreased osteointegration. There are many mechanisms through which AGEs determine tissue damage: they suppress the production of collagen by the gingival and the periodontal ligament fibroblast [30, 31]; they contribute to delayed wound healing [32] and they inhibit the phenotypic expression of osteoblasts [33], while stimulating osteoclastogenesis with consequent bone resorption [34, 35]. In this regard, the diabetic bone is characterized by a reduced turnover that sees the reabsorption process prevail over the newly affixed one, with reduced mineral bone density [8], increased tendency to fracture [9], and poor bone healing and impaired bone regeneration potential [10–12]. Impaired wound healing is a well-known consequence of diabetes, which may be related to the local growth factors that influence cell migration, proliferation, and phenotypic expression [36]. For this reason, it can be suggested, although no direct data are available, that diabetics who are not optimally controlled may undergo an altered healing response after surgical procedures performed to correct osseous defects. Improved metabolic control is currently the only practical approach to managing this risk factor. Given current knowledge, it can be supposed that the clinical significance of the disease relative to its impact on the healing response will be a function of the control of glucose metabolism [37].

In our study, the EHS (early wound healing score) in each group showed a significant improvement from week 1 to week 3, with almost all of the patients reaching a final score of ten points, representing the best healing. In addition, the glycemic level seems not to have influenced the healing process outcome. Healing takes place following a well-organized chronology of biological events that are crucial for the quality of the final repair of wounded tissues [38]. In particular, the first postoperative week appears to be critical for the maintenance of wound stability. Wound healing should be monitored to identify early signs that may be related to healing complications. Such findings might be associated with problems in different surgical procedures, and surgeons should be aware of these problems to consider prompt interventions [39–42]. In our study, 2 patients presented biological complications during the first 3 weeks after surgery. All of the complications were cases of wound dehiscence, which were treated by local disinfection, and all affected patients recovered completely after 2–3 weeks. The complication rate of the present study is in agreement with that of the study by von Arx and Buser (2006) [43], in which the main complications were small membrane exposures that went to reepithelialization within 2–4 weeks. On the other hand, a systematic review of Lim et al. (2018) [44] concludes that soft tissue complications after GBR are common, appearing in 18.6% of cases.

Impaired osseointegration could be one of the results of hyperglycemia's effect on the bone mineralization and remodelling process [45, 46], with reduced bone-to-implant contact (BIC) being reported in the literature [46]. However, it seems that good glycemic control based also on the administration of insulin improves osseointegration and implant survival [26], even if decreased BIC may be observed in comparison to nondiabetic subjects [19]. In the present study, the survival rate, meaning whether the implant was still physically in the mouth or had been removed [47], was 98%.

Furthermore, a statistically significant variation regarding the height and width of the peri-implant bone defects was observed after the procedure, with a decrease for both parameters after 6 months. No statistically significant differences were observed between the HbA1c levels and the variations of VDH and DW after surgery. However, it should be considered that the best clinical outcomes, regarding the variables ΔVDH and ΔDW, occurred in patients with lower HbA1c levels. Even subjects with controlled diabetes (HbA1c < 7%) have shown a significant reduction in the height and width of the bone defects. In our study, 82.4% of the patients achieved a residual height and width of the bone defect equal to 0 mm at *T*_1_.

These findings are in agreement with those from a series of previous studies: histometric data on GBR in diabetic conditions [48, 49] confirm the potential of the GBR application to promote bone regeneration, even in the presence of uncontrolled experimental diabetes. Retzepi et al. [14] and Donos et al. [50] come to the same conclusion, adding that the diabetic status is associated with impaired healing and increased complications, which are improved when metabolic control via systemic insulin is performed. Retzepi et al. also underline that, following GBR execution in combination with implant placement, the type of the contact between the augmented diabetic bone and the implant surface is similar to that of the healthy bone.

Naujokat et al. (2016) [26], in a systematic review of the literature, say that no evidence was found that bone augmentation procedures such as guided bone regeneration and sinus lift provide higher complication and failure rates in patients with well- to fairly well-controlled diabetes.

In the present study, optimal results were recorded also for the periodontal parameters during the follow-up period, outlining that patient selection and the use of specific postsurgical cleansing protocols may play a key role in the healing process after an augmentation procedure in order to facilitate wound healing and closure [50, 51].

Long-term peri-implant success depends primarily on oral hygiene care. This success is inculcated within the patients by general practitioners and specialists that reinforce optimal oral hygiene maintenance, which actually prolongs treatment success.

Furthermore, in all the clinical conditions, the use of digital technologies in the preoperative phase (for planning the implant position and assessing the required bone volume to regenerate) as well as during the surgery (for customizing the membrane or the mesh) may help the clinician to improve the clinical results and avoid postoperative complications related to the operator [52].

The study presents some limitations: primarily its retrospective nature and the small sample of participants, in addition to a short-term follow-up and the absence of analysis of cellular or molecular factors, in order to elucidate the mechanism of bone healing. Surely, further large-scale studies with a longer follow-up and histological examinations would reinforce the significance of these results.

## 5. Conclusions

Simultaneous guided bone regeneration seems to be a feasible surgical procedure for the treatment of peri-implant dehiscences in controlled type 2 diabetic patients with HbA1c levels below 7%. The sample size of the present retrospective study consisted of a limited number of subjects with a short-term follow-up. Thus, prospective long-term study should be conducted to verify these findings.

## Figures and Tables

**Table 1:** Patients' demographics and clinical features.

	Total (*n* = 34)		No success (*n* = 6)	Success (*n* = 28)	*p* value
*Gender*					
M (*n*, %)	19	55.9	4 (66.7)	15 (53.6)	0.672
F (*n*, %)	15	44.1	2 (33.3)	13 (43.4)	
*Age (mean* ± *SD*)	69.56 ± 8.2		72.5 ± 7.74	68.93 ± 8.3	0.413
*BMI (mean* ± *SD*)	26.9 ± 2.5		28.2 ± 1.8	26.6 ± 2.5	0.145
*Osteopenia*					
No (*n*, %)	26	76.5	5 (83.3)	21 (75.0)	0.662
Yes (*n*, %)	8	23.5	1 (16.7)	7 (25.0)	
*Periodontal prophylaxis*					
No (*n*, %)	14	41.2	4 (66.7)	10 (35.7)	0.162
Yes (*n*, %)	20	58.8	2 (33.3)	18 (64.3)	
*BOP (bleeding on probing)*					
No (*n*, %)	27	79.4	5 (83.3)	22 (78.6)	0.793
Yes (*n*, %)	7	20.6	1 (16.7)	6 (21.4)	
*Glycemic level*					
1	14	41.2	1 (16.7)	13 (46.4)	0.405
2	8	23.5	2 (33.3)	6 (21.4)	
3	12	35.3	3 (50.0)	9 (32.1)	
*N. implants*					
1	21	61.8	1 (16.7)	20 (71.4)	0.042
2	10	29.4	4 (66.7)	6 (21.4)	
3	3	8.8	1 (16.7)	2 (7.1)	
*Infrabony defect height*					
0	6	17.6	5 (83.3)	1 (3.6)	<0.0005
1	7	20.6	1 (16.7)	6 (21.4)	
2	16	47.1	0 (0.0)	16 (57.1)	
3	5	14.7	0 (0.0)	5 (17.9)	
*VDHT*_0_ *(vertical defect height) (mean* ± *SD*)	2.74 ± 0.828		3 ± 1.09	2.68 ± 0.77	0.644
*DWT*_0_ *(defect width) (mean* ± *SD*)	3.35 ± 0.950		3.50 ± 1.049	3.32 ± 0.945	0.708
*HDDT*_0_ *(horizontal defect depth) (mean* ± *SD*)	1.59 ± 0.957		0.17 ± 0.408	1.89 ± 0.737	
*KTT*_0_ *(keratinized tissue) (mean* ± *SD*)	3.94 ± 0.7		4.00 ± 0.894	3.93 ± 0.716	0.878
*VDHT*_1_(*mean* ± *SD*)	0.24 ± 0.554		1.33 ± 0.516	0 ± 0	<0.0005
*DWT*_1_(*mean* ± *SD*)	0.24 ± 0.554		1.33 ± 0.516	0 ± 0	<0.0005
*KTT*_1_(*mean* ± *SD*)	3.38 ± 0.739		3.0 ± 0.894	3.46 ± 0.693	0.297
*EHS week 1 (early wound healing score)*					
2	2	5.9	2 (33.3)	0 (0.0)	0.017
6	7	20.6	1 (16.7)	6 (21.4)	
7	20	58.8	2 (33.3)	18 (64.3)	
8	5	14.7	1 (16.7)	4 (14.3)	
*EHS week 3 (early wound healing score)*					
8	2	5.9	2 (33.3)	0 (0.0)	0.004
9	3	8.8	1 (16.7)	2 (7.1)	
10	29	85.3	3 (50.0)	26 (92.9)	
*Complications*					
No (*n*, %)	32	94.1	4 (66.7)	28 (100.0)	0.002
Yes (*n*, %)	2	5.9	2 (33.3)	0 (0.0)	

**Table 2:** Vertical defect height changes from *T*_0_ to *T*_1_, measured in mm.

Descriptive statistics				
	Glycemic level	Mean	Std. deviation	*N*
VDH *T*_0_ (vertical defect height)	1	2.50	0.760	14
	2	2.88	0.991	8
	3	2.92	0.793	12
	Total	2.74	0.828	34
VDH *T*_1_	1	0.07	0.267	14
	2	0.50	0.926	8
	3	0.25	0.452	12
	Total	0.24	0.554	34

**Table 3:** Defect width changes from *T*_0_ to *T*_1_, measured in mm.

Descriptive statistics				
	Glycemic level	Mean	Std. deviation	*N*
DW *T*_0_ (defect width)	1	3.50	0.941	14
	2	3.38	1.061	8
	3	3.17	0.937	12
	Total	3.35	0.950	34
DW *T*_1_	1	0.07	0.267	14
	2	0.38	0.744	8
	3	0.33	0.651	12
	Total	0.24	0.554	34

## Data Availability

The datasets used and/or analysed during the current study are available from the corresponding author on reasonable request.
